# Comparison of Lignocellulose Nanofibrils Extracted from Bamboo Fibrous and Parenchymal Tissues and the Properties of Resulting Films

**DOI:** 10.3390/polym16131829

**Published:** 2024-06-27

**Authors:** Xiaofeng Zhang, Jingpeng Li, Gege Bao, Daochun Qin, Xiaobei Jin

**Affiliations:** 1 Research Institute of Bamboo and Rattan Biomass and New Materials, International Centre for Bamboo and Rattan, Beijing 100102, China; 2 Key Laboratory of High Efficient Processing of Bamboo of Zhejiang Province, Engineering Technology Research Center for Building and Decorating Materials of Bamboo State Forestry Administration, China National Bamboo Research Center, Hangzhou 310012, China; 3 Sanya Research Base, International Centre for Bamboo and Rattan, Sanya 572000, China

**Keywords:** bamboo, parenchymal tissue, lignocellulose nanofibrils, metal organic framework

## Abstract

Bamboo is composed of thick-walled fibrous tissue and thin-walled parenchymal tissue. To compare the energy consumption of preparing lignocellulose nanofibrils (LCNF) from these bamboo tissues, the crystallinity, sol. viscosity, morphology and mechanical properties of LCNF at different preparation stages were characterized in detail. It required at least nine homogenization cycles for dissociating the fibrous tissue, but only six cycles for the parenchymal tissue. The average diameter of LCNF isolated from fibrous and parenchymal tissues was 45.1 nm and 36.2 nm, respectively. The tensile strength of the LCNF film prepared from parenchymal tissue reached 142.46 MPa, whereas the film from fibrous tissue reached only 122.82 MPa. Additionally, a metal organic framework (MOF) was used to produce MOF-LCNF film with enhanced UV protection and antibacterial properties. The results indicated that the energy consumption for preparing LCNF from parenchymal tissue is significantly lower than that for preparing LCNF from fibrous tissue. This study offers a low-cost and eco-friendly method for preparing LCNF, promoting the precise utilization of different tissues from bamboo based on their unique characteristics.

## 1. Introduction

There is growing interest in utilizing renewable lignocellulosic biomass as an alternative to fossil resources [1]. Cellulose nanofibril (CNF) has been widely used in various fields due to its excellent mechanical properties, degradability and biocompatibility [2].

Nanocellulose is generally extracted from wood-based materials, such as wood, bamboo and cotton [3,4,5]. Bamboo has emerged as an appealing raw material for CNF production because of its rapid growth rate, high yield and large cellulose content [6]. Bamboo is composed of fibrous and parenchymal tissues, each with distinct cell structures and chemical compositions [7]. The morphology and structure of microfibril derived from the same raw material vary with the selected parts. For instance, Okahisa reported that CNF obtained from different parts of oil palm exhibited distinct mechanical and thermal properties [8]. Previous studies have shown that the CNF derived from bamboo fibrous and parenchymal tissues shared similar crystallinity but differed in tensile strength [9,10,11]. However, there is currently a lack of comparative research regarding the energy consumption for preparing CNF from these bamboo tissues. The cellulose molecule chains in fibrous tissue are arranged more closely than those in parenchymal tissue [12], potentially affecting the difficulty of microfibril depolymerization during the preparation of CNF. In order to promote the precise utilization of different tissues from bamboo, it is necessary to study the differences in the preparing CNF from bamboo fibrous and parenchymal tissues.

CNF was traditionally prepared from sulfuric acid bleached pulp, which led to the waste of lignin and hemicellulose [13]. Some studies have reported the production of lignocellulose nanofibrils (LCNF) by retaining partial lignin [14]. LCNF has the benefits of a high yield of up to 77.2%, low cost and less environmental impact [15]. The residual lignin in nanocellulose can also endow the products with synergistic functions. The films made from LCNF showed lower water uptake and better wet mechanical properties compared to those made from CNF without lignin [16,17]. Moreover, lignin is rich in phenylpropane units, phenols and ketones, which can absorb ultraviolet radiation [18]. Therefore, LCNF can be used to prepare cellulose nanomaterials for UV protection [19,20].

Film is the most common application form of nanocellulose. It is known for its high strength, excellent barrier properties and biodegradability, making it suitable for use as an eco-friendly material in the packaging field [21,22,23]. However, the cellulose in the nanocellulose film is susceptible to microbial erosion and cannot meet the antibacterial requirements of packaging materials. To solve this problem, some studies have developed natural fiber composites for improving the antibacterial property by incorporating antibacterial materials, such as essential oils, nano sliver and a metal organic framework (MOF) [24,25,26,27]. The MOF can inhibit bacterial growth and reproduction by disrupting the cell membrane of bacteria [28]. With rich variety and good biocompatibility, the MOF demonstrates great potential for application in antibacterial materials [29,30,31].

Here, bamboo fibrous and parenchymal tissues were separated by a simple method, without destroying the cell structure. Subsequently, LCNF based on bamboo fibrous and parenchymal tissues was prepared by acid hydrolyses combined with high-pressure homogenization. The effect of the cell structure on energy consumption during nanocellulose preparation at different processing stages was analyzed by characterizing the morphology, crystallinity, and mechanical properties of the LCNF. Finally, a Zeolitic Imidazolate Framework (ZIF-8) was added into the LCNF gel to obtain a MOF-LCNF film with enhanced UV protection and antibacterial properties.

## 2. Materials and Methods

### 2.1. Materials

Moso bamboo (Phyllostachys edulis) was obtained from Hangzhou, Zhejiang Province, China. Firstly, the bamboo wax and tabasheer were removed by a physical method. All the samples were ground using a small plant grinder (GX-10B, Zhejiang Gaoxin Co., Ltd., Yongkang, China) and then passed through a 30-mesh and a 60-mesh sieve in turn. Maleic acid (C_4_H_4_O_4_), sodium chlorite (NaClO_2_) and potassium hydroxide (KOH) were purchased from Shanghai Aladdin Reagent Co., Shanghai, China. Zeolitic Imidazolate Frameworks (ZIF-8, C_8_H_12_N_4_.Zn) with a diameter of approximately 800 nm were purchased from Shanghai Sigma-Aldrich Co., Shanghai, China.

### 2.2. Physical Separation of Bamboo Fibrous and Parenchymal Tissues

**Figure 1:**
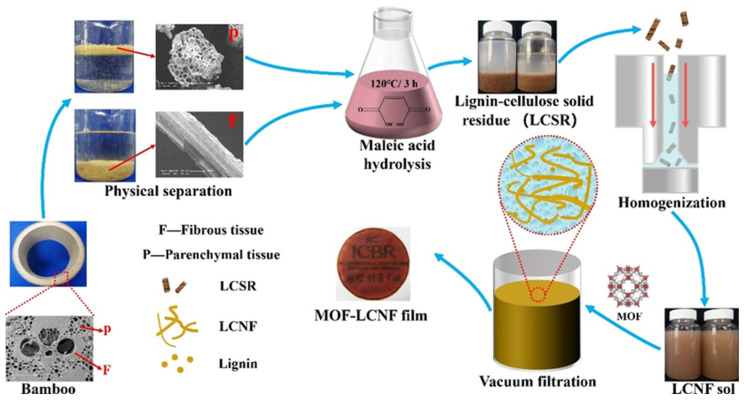
Scheme of preparing MOF-LCNF film from bamboo fibrous and parenchymal tissues.

Bamboo powder between 30–60 mesh was soaked in deionized water for 2 min. Due to the difference in density, the fibrous tissue (F) sinks to the bottom while the parenchymal tissue (P) floats on the surface. This density difference can be visually expressed by their micro morphology (as shown in Figure 1): there is a large cell cavity in the parenchymal cell, but the fibrous cell is mainly composed of solid substance. Therefore, the cell wall–cavity ratio of parenchymal tissue is significantly lower than that of fibrous tissue. The SEM pictures proved that the fibrous and parenchymal tissues were separated efficiently.

### 2.3. Preparation of LCNF

Acid hydrolysis was conducted to remove a part of the lignin and hemicellulose in the raw materials [32]. An amount of 20 g of fibrous or parenchymal tissue mixed with 200 mL of a maleic acid solution (60 wt%) was maintained at 120 °C for 3 h. The mixture was constantly stirred at 300 rpm during hydrolysis. The products of maleic acid hydrolysis were mainly composed of lignin and cellulose solid residue (F-LCSR, P-LCSR). After washing, the LCSR samples were homogenized with a concentration of 0.5 wt% to prepare the LCNF. The LCNF samples derived from fibrous and parenchymal tissues were named F-LCNF and P-LCNF, respectively. The LCNF from fibrous tissue passing through the homogenizer 1, 2, 4, 6, 9, 12 and 15 times were named FM1, FM2, FM4, FM6, FM9, FM12 and FM15, respectively, whereas the LCNF from parenchymal tissue were named PM1, PM2, PM4, PM6, PM9, PM12 and PM15, respectively. Additionally, the CNF without lignin was also prepared from fibrous and parenchymal tissues (F-CNF and P-CNF) according to a previous method [33].

### 2.4. Preparation of LCNF and MOF-LCNF Films

LCNF film: 200 mL of LCNF sol. was vacuum-filtered using a filter membrane to obtain the LCNF gel sheet. Subsequently, the sheet was pressed under a heavy object loading of 10 kg. Finally, the sheet was dried at ambient temperature for 5 days.

MOF-LCNF film: ZIF-8 was mixed with 200 mL LCNF sol. and stirred for 3 h at 400 rpm using a magnetic stirrer. The LCNF sol. with ZIF-8 was filtered and dried using the same method as that used for the LCNF film. The films were labeled as MOF-LCNF-15%, MOF-LCNF-10% and MOF-LCNF-5%, based on the mass proportion of ZIF-8.

### 2.5. Characterization

Morphology analysis was conducted by a scanning electron microscope (SEM, Philips B.V., Eindhoven, The Netherlands) at 7 KV. The samples were freeze-dried and then sprayed with gold to enhance conductivity.

The contents of α-cellulose and lignin were characterized by a method involving sulfuric acid hydrolysis [34,35]. The hemicellulose content was calculated from the difference between the holocellulose and the α-cellulose contents [36].

All the samples were analyzed using Fourier transform infrared (FTIR) spectroscopy (Thermo Fisher Scientific, Waltham, MA, USA) around 4000–500 cm^−1^.

The X-ray diffraction (XRD) patterns of the samples were recorded by a diffractometer (PANalytical B.V., Almelo, The Netherlands) around 5–40°, at 40 kV and 40 mA.

AFM samples were dried on a sample platform and observed using an Atomic Force Microscope (Bruker AFM, Santa Barbara, CA, USA).

The viscosity of 0.5 wt% LCNF sol. was analyzed at ambient temperature using a rotary viscometer. The change of viscosity with time was recorded until stable.

A zeta potential analyzer (DT-300/310, Quantachrome, Boynton Beach, FL, USA) was used to measure the zeta potential of the LCNF sol. The sample was diluted to a concentration of 0.5 g/L. The average value was calculated using three samples in each group.

Thermal stability was conducted using a thermogravimetric analyzer (Q500, TA Instruments, New Castle, DE, USA) from ambient temperature to 600 °C at a nitrogen rate of 20 mL/min.

The optical properties of the LCNF and CNF films were investigated using a Varian Cary 300 UV–vis. spectrometer (DQI2010, Craic, San Dimas, CA, USA) around 0–800 nm.

Tensile tests were measured by a tensile tester (Instron 5848, Instron, Norwood, MA, USA). The average value of tensile strength and Young’s modulus were calculated from the average of 5 tests

The antibacterial property was measured as follows: Aerosols containing *Escherichia coli*, with diameters ranging from 1 to 5 μm, were generated using a bacterial suspension with 106 colony-forming units (CFU) per milliliter. These aerosols served as a model for infection. The MOF-LCNF film was then exposed to a flow of aerosols at a rate of 0.3 mL per minute for a duration of 5 min. Subsequently, the MOF-LCNF film underwent thorough washing with 20 mL of saline solution. The concentrations of bacteria in the eluant were measured according to the standard plate count method. Each experiment was repeated for 3 times.

## 3. Results and Discussion

### 3.1. Chemical Composition and Yield of LCSR Samples

The chemical composition and yield after maleic acid hydrolysis are shown in Table 1. The fibrous tissue contained less hemicellulose but more cellulose than the parenchymal tissue. This difference can be attributed to their roles in bamboo growth. Parenchymal tissue provides nutrition for growth by organic substance and sugar, while fibrous gives mechanical support [12]. After acid hydrolysis, the cellulose content of the LCSR from fibrous and parenchymal tissues increased to 62.65% and 60.1%, indicating that most hemicellulose has been removed. The LCSR samples derived from fibrous and parenchymal tissues exhibited similar chemical composition. Additionally, the high yield (65.75% and 61.61%) came from the retention of lignin.

### 3.2. FTIR Analysis

**Figure 2:**
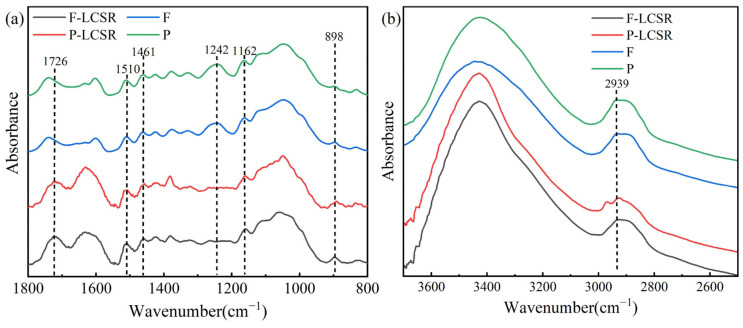
FTIR spectra of raw materials and LCSR samples at wavenumber of 800−1800 nm (**a**) and 2500−3700 nm (**b**).

Figure 2 exhibits the FTIR spectra of raw materials and LCSR. The absorption bands derived from cellulose structures at 1162 and 898 cm^−1^ can be seen in all spectra [37]. The absorption bands at 1510 and 1461 cm^−1^ derived from lignin [38] were especially obvious in the LCSR samples. The absorption band at 1242 cm^−1^, attributed to the glucuronic acid of hemicellulose, showed diminished intensity after acid hydrolysis, which confirmed the removal of hemicellulose [39]. In addition, an absorption band at 1726 cm^−1^ associated with the carbonyl group can be only seen in the LCSR samples. The change of absorption band at 2939 cm^−1^ also demonstrates that maleic acid introduced carboxyl groups to the LCSR [32].

### 3.3. Effect of Homogenization on the Crystallinity of LCNF

**Figure 3:**
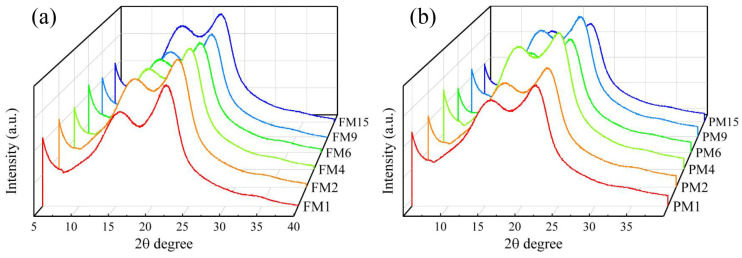
XRD spectra of LCNF at different homogenization stages from fibrous tissue (**a**) and parenchymal tissue (**b**).

All the samples exhibited diffraction peaks at 2θ = 16.5° and 22.4°, which was typical for the cellulose I pattern in natural plants (Figure 3). The crystallinity was calculated based the method of the Segal Crystallinity Index [40]. The crystallinity (42.55% and 37.21% for F-LCSR and P-LCSR, respectively) was lower than that of the samples derived from the bamboo bleached pulp [41]. The lignin in the samples contributed a high proportion of amorphous regions. After several cycles of homogenization, a significant decrease in crystallinity can be observed in the LCNF samples. A high-intensity shear and impact force destroyed the hydrogen bonds between the cellulose chains, resulting in a sparse arrangement [42]. It is noteworthy that the crystallinity of the parenchymal tissue decreased more significantly than that of the fibrous tissue after one cycle of homogenization (Table S1), and during the whole process, the relative crystallinity of the parenchymal tissue was always lower than that of the fibrous tissue due to the difference in cell wall structure.

### 3.4. Effect of Homogenization on the Viscosity of LCNF Sol.

**Figure 4:**
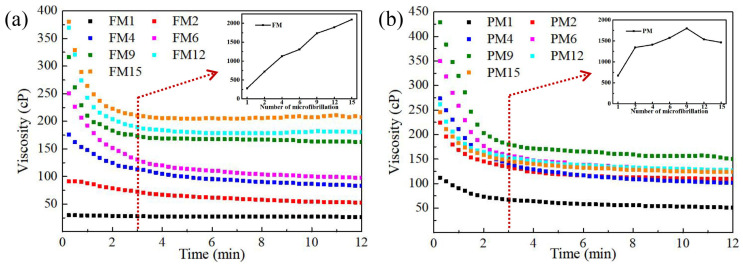
Viscosity of LCNF at different homogenization stages from fibrous tissue (**a**) and parenchymal tissue (**b**).

The viscosity depends strongly on the cross-linking and mechanical entanglement of the fibrils and polymer chains, which reflects the degree of depolymerization of microfibrils [43]. The viscosity of the LCNF sol. decreased gradually until stable because of shear-thinning behavior (Figure 4). In order to compare the depolymerization degree, the viscosity after 3 min was selected as stable viscosity. A significant increase can be observed during the initial stages of homogenization. A high-pressure homogenization accelerated the depolymerization of microfibrils and led to a high length–diameter ratio [44]. Further homogenization, however, disrupts these cellulose nanofibrils into smaller fragments with a low length–diameter ratio. After nine cycles of homogenization, the viscosity of the LCNF sol. derived from the parenchymal tissue reached the maximum, and then began to decrease. Interestingly, this trend did not appear in the LCNF sol. from fibrous tissue during 15 cycles of homogenization. These results suggest that the parenchymal tissue was more sensitive to mechanical treatment compared to the fibrous tissue.

### 3.5. Morphology Analysis

**Figure 5:**
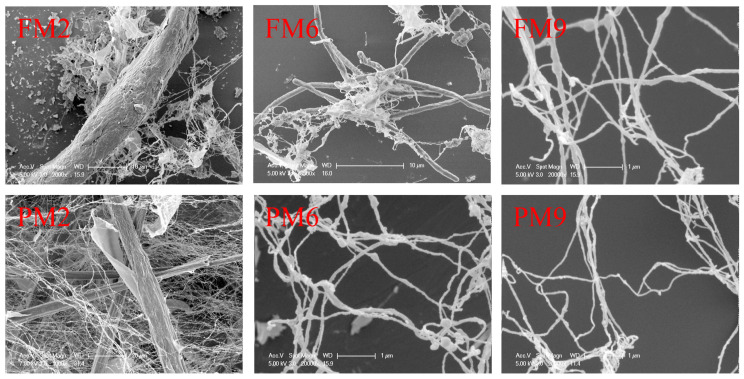
Morphology of LCNF at different homogenization stages from fibrous and parenchymal tissues.

Figure 5 shows the morphological change in the LCNF samples during homogenization. The shear force generated by high-pressure homogenization can effectively destroy the binding between fibrils. After two cycles of homogenization, the fibrous and parenchymal tissues were not completely dissociated, and large-diameter fiber bundles could be clearly observed. With six cycles of homogenization, the parenchymal tissue was completely dissociated into nanofibrils less than 100 nm in diameter, while some fiber bundles remained in the fibrous tissue. The complete isolation of nanofibrils from fibrous tissue required nine cycles of homogenization. A zeta potential absolute value above 30 is generally indicative of system stability [45], a finding supported by the observations in Table S2. It took at least nine cycles of homogenization to form a stable sol. for the fibrous tissue, while at most six cycles for the parenchymal tissue.

**Figure 6:**
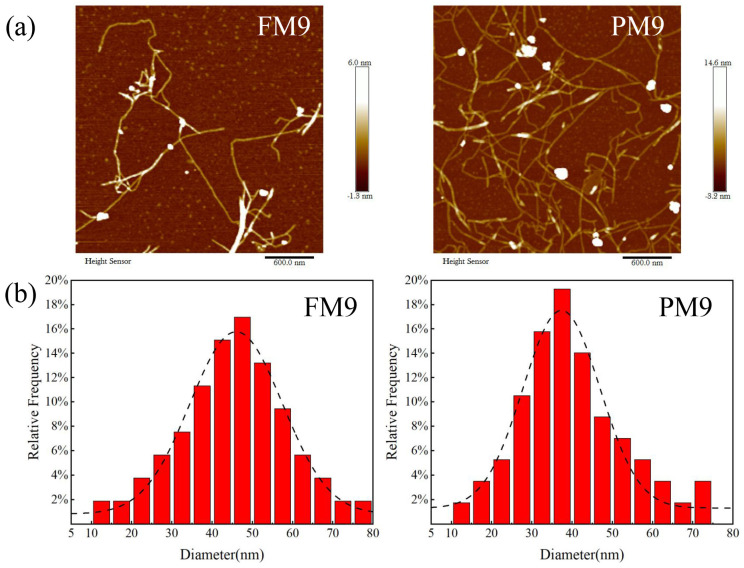
AFM images (**a**) and diameter distribution (**b**) of LCNF after 9 cycles of homogenization.

The diameter distribution of the LCNF after nine cycles of homogenization is shown in Figure 6. The average diameter of the LCNF prepared from fibrous and parenchymal tissues was 45.1 nm and 36.2 nm, respectively. Furthermore, the diameter of the LCNF obtained from parenchymal tissue exhibited a narrow distribution concentrated in 30–40 nm. It was observed that fibrous tissue required ultrasonication or grinding before homogenization to prevent the pipes of the homogenizer from clogging. However, the parenchymal tissue did not require this pretreatment.

According to these results, it can be seen that more mechanical energy may be required to dissociate fibrils in the fibrous tissue, compared to the parenchymal tissue. Previous research demonstrated that the energy consumption required to prepare nanocellulose from plant fibers depends largely on their chemical composition, cell morphology and cell wall structure [8]. In this study, the chemical composition of fibrous and parenchymal tissues was found to be similar after acid hydrolysis. Therefore, the difference in energy consumption mainly came from their cell structures. The parenchymal tissue, considered as feeble cells, has a much thinner cell wall and a larger cell cavity than the fibrous tissue [46]. In addition, compared to the fibrous tissue, the cellulose molecule chains wind less tightly in the parenchymal tissue [12], making it easier for the parenchymal tissue to destroy the cell wall and extract nanofibrils.

### 3.6. Thermal Stability

**Figure 7:**
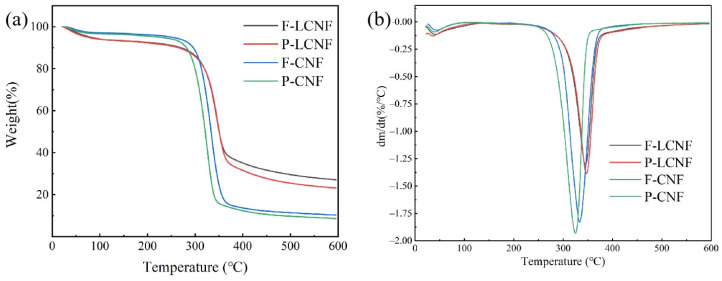
TGA curves (**a**) and corresponding DTG curves (**b**) of samples after 9 cycles of homogenization.

LCMF samples after nine cycles of homogenization were selected to characterize the thermal stability (Figure 7). The weight loss between 50–110 °C was attributed to water evaporation. Notably, there was no significant difference in the decomposition temperature observed between the LCNF samples derived from the fibrous and parenchymal tissues. The onset temperature and maximum temperature of the LCNF was higher than those of the CNF samples due to the high residual lignin (more than 20%) and the covalent linkage between cellulose and lignin [47,48]. Lignin, containing diverse aromatic groups, decomposes over a wider temperature range compared to cellulose and hemicellulose. Consequently, the LCNF demonstrates promise for applications in composites that require superior thermal stability.

### 3.7. Mechanical Properties of Films

**Figure 8:**
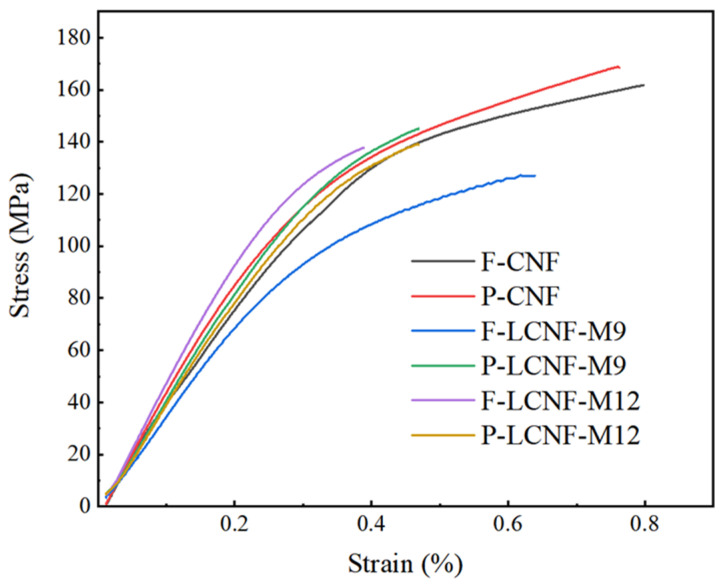
Tensile strength of LCNF and CNF films from fibrous and parenchymal tissues.

The tensile strength and Young’s modulus were calculated according to the stress–strain curves (Figure 8). Compared to the CNF films, the LCNF films exhibited weaker mechanical properties. The mechanical property of the CNF film was positively correlated with the hydrogen bonding between microfibrils [49]. The lignin in the LCNF prevented the formation of hydrogen bonding. For the samples obtained from the fibrous tissue, the tensile strength was 122.82 and 135.49 MPa for F-LCNF-M9 and F-LCNF-M12, respectively (Table S3). Homogenization caused the separation of fibrils, exposing the hydroxyl groups on the surface. However, for samples from the parenchymal tissue, the tensile strength after 9 and 12 cycles was 142.46 and 136.76 MPa, respectively. Excessive homogenization led to a decrease in tensile strength, as excessive mechanical treatment reduced only the length of the fibrils, not the diameter, affecting the intertwining between fibrils. Under the same conditions, the mechanical strength of the LCNF film from the parenchymal tissue was always higher than that from the fibrous tissue, possibly due to the lower crystallinity and smaller diameter.

### 3.8. Optical Property of Films

**Figure 9:**
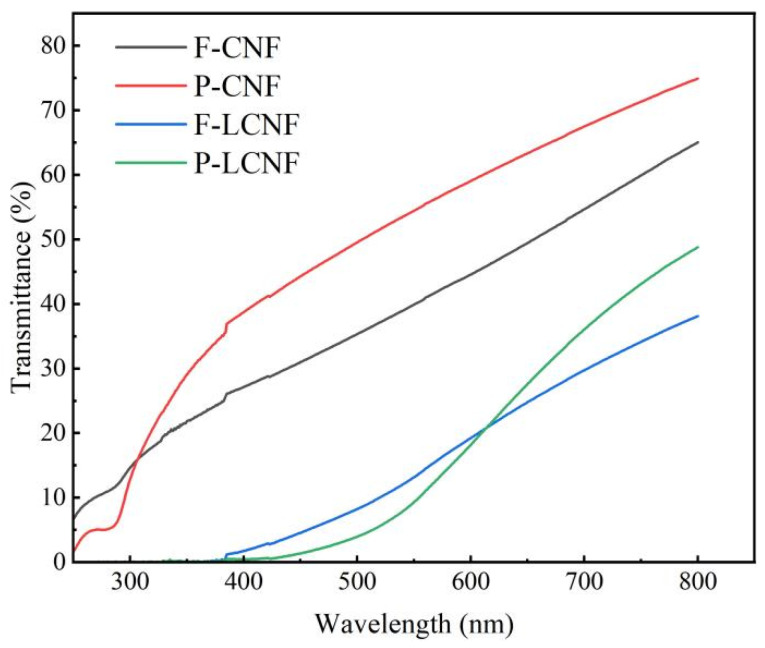
UV protection property of CNF and LCNF films.

The UV protection properties of the LCNF and CNF films are displayed in Figure 9. The CNF film showed a high transparency in the ultraviolet region below 400 nm. Conversely, the LCNF film blocked almost all the UV-B (280–320 nm) and over 90% of UV-A (320–400 nm), which approached the UV protection property of the polymer film [20]. The phenolic and ketone groups in lignin, which can adsorb UV, gave the LCNF film excellent UV protection properties. Furthermore, the transmittance of the film prepared from F-LCNF was significantly lower than that from P-LCNF. This difference could be explained by the fact that the average diameter of F-LCNF was higher than that of P-LCNF.

### 3.9. Antibacterial Activity of MOF-LCNF Films

**Figure 10:**
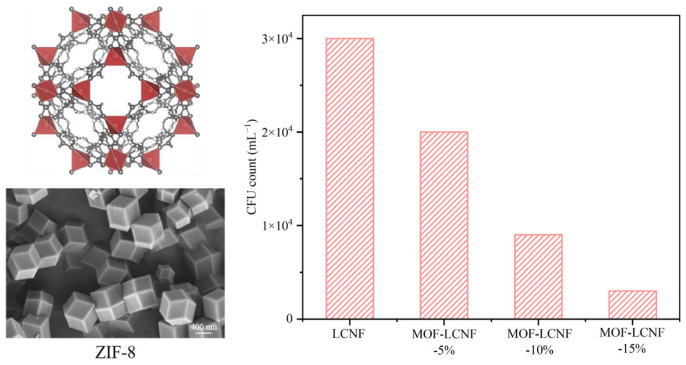
SEM of ZIF-8 and residual levels of *Escherichia coli* on MOF-LCNF film.

The MOF possesses distinctive antibacterial properties, as its surface metal ions and organic ligands can interact with bacterial cell walls, resulting in membrane damage and cell death [28]. By integrating MOF into the LCNF film, its antibacterial properties can be fully harnessed, resulting in improved antibacterial properties (Figure 10). As the mass ratio of the MOF increased, the concentration of live bacteria significantly decreased, with a minimum of 3600 CFU/mL, compared to 30,000 CFU/mL in the LCNF film without a MOF. An excellent bactericidal property highlights the potential for application in packing materials that require antibiotic action.

## 4. Conclusions

In this study, LCNF with a high lignin content was produced from bamboo fibrous and parenchymal tissues by maleic acid hydrolysis combined with high-pressure homogenization. After the acid hydrolysis, the fibrous and parenchymal samples exhibited a similar chemical composition (about 30% of lignin) and a yield exceeding 60%. Notably, the fibrous tissue necessitated more energy during mechanical processing compared to the parenchymal tissue. To isolate the nanofibrils completely, six cycles of homogenization were required for the parenchymal tissue, but at least nine cycles for the fibrous tissue. Under the same conditions, the LCNF derived from parenchymal tissue displayed lower crystallinity, a smaller diameter and better mechanical properties. The observed distinctions can be attributed to the cell structure. In addition, the presence of lignin and the MOF enhanced the UV protection and antibacterial properties of the LCNF film. This investigation exemplifies an enhanced strategy for the precise utilization of bamboo fibrous and parenchymal tissues.

## Figures and Tables

**Table 1:** Chemical composition and yield of LCSR samples after maleic acid hydrolysis.

Samples	α-Cellulose (%)	Hemicellulose (%)	Klason Lignin (%)	Total Yield (%)
F	45.14 ± 1.42	28.76 ± 1.19	23.27 ± 0.88	-
F-LCSR	62.65 ± 3.14	4.31 ± 1.44	31.38 ± 1.25	65.75 ± 2.16
P	34.62 ± 1.25	39.14 ± 1.38	21.33 ± 1.07	-
P-LCSR	60.10 ± 2.17	5.24 ± 3.04	30.71 ± 2.06	61.61 ± 3.33

## Data Availability

The original contributions presented in the study are included in the article/Supplementary Materials, further inquiries can be directed to the corresponding author/s.

## Supplementary Materials

The following supporting information can be downloaded at: https://www.mdpi.com/article/10.3390/polym16131829/s1, Table S1: Crystallinity of samples at different homogenization stages; Table S2: Zeta potential of samples at different homogenization stages; Table S3: Tensile strength and elastic modulus of samples at different homogenization stages.
