# Supplementary material for: Comparison of Lignocellulose Nanofibrils Extracted from Bamboo Fibrous and Parenchymal Tissues and the Properties of Resulting Films
Source: Polymers (Basel). 2024 Jun 27;16(13):1829. doi: 10.3390/polym16131829 (PMC11243877; doi:10.3390/polym16131829)
Supplement: Supplementary file 1 [file polymers-16-01829-s001.zip › polymers-3071984-supplementary.pdf]

Supplementary data

Comparison of Lignocellulose Nanofibrils Extracted from Bamboo Fibrous and Parenchymal Tissues and the Properties of Resulting Films

Xiaofeng Zhang [1], Jingpeng Li [2], Gege Bao [1], Daochun Qin [3,*] and Xiaobei Jin [1,*]

Table captions

Table S1 Crystallinity of samples at different homogenization stages.

Table S2 Zeta potential of samples at different homogenization stages.

Table S3 Tensile strength and elastic modulus of samples at different homogenization stages.

Table S1 Crystallinity of samples at different homogenization stages.

Samples	Crystallinity (%)	Samples	Crystallinity (%)
FM1	27.66	PM1	18.89
FM2	20.54	PM2	18.26
FM4	20.72	PM4	16.20
FM6	19.08	PM6	17.46
FM9	24.29	PM9	18.08
FM15	17.33	PM15	15.07

Table S2 Zeta potential of samples at different homogenization stages.

Samples	Zeta potential (mV)	Samples	Zeta potential (mV)
FM2	-15.5	PM2	-17.6
FM6	-27.3	PM6	-30.8
FM9	-30.9	PM9	-32.8
FM12	-31.2	PM12	-33.7

Table S3 Tensile strength and elastic modulus of samples at different homogenization stages.

Samples	Tensile strength (MPa)	Young's modulus (GPa)
F-CNF	172.21	12.50
P-CNF	178.07	12.91
F-LCNF-M9	122.82	7.46
P-LCNF-M9	142.46	11.26
F-LCNF-M12	135.49	10.19
P-LCNF-M12	136.76	10.81
